# Integrated Lipidomic and Transcriptomic Analysis Reveals Phospholipid Changes in Somatic Embryos of *Picea asperata* in Response to Partial Desiccation

**DOI:** 10.3390/ijms23126494

**Published:** 2022-06-10

**Authors:** Juanjuan Ling, Yan Xia, Jiwen Hu, Tianqing Zhu, Junhui Wang, Hanguo Zhang, Lisheng Kong

**Affiliations:** 1 State Key Laboratory of Tree Genetics and Breeding, Key Laboratory of Tree Breeding and Cultivation of State Forestry and Grassland Administration, Research Institute of Forestry, Chinese Academy of Forestry, Beijing 100091, China; 18829351306@163.com (J.L.); yansummer@swu.edu.cn (Y.X.); hujiwen020@126.com (J.H.); 2 Key Laboratory of Horticulture Science for Southern Mountains Regions of Ministry of Education, College of Horticulture and Landscape Architecture, Southwest University, Chongqing 400715, China; 3 State Key Laboratory of Tree Genetics and Breeding, Northeast Forestry University, Harbin 150040, China; hanguo.zhang@nefu.edu.cn; 4 Department of Biology, Centre for Forest Biology, University of Victoria, Victoria, BC V8P 5C2, Canada; lkong@uvic.ca

**Keywords:** *Picea asperata*, somatic embryo, partial desiccation treatment, phospholipids, phospholipid acid

## Abstract

Partial desiccation treatment (PDT) is an effective technology for promoting the germination and conversion of conifer somatic embryos (SEs). PDT, as a drought stress, induces intensive physiological responses in phospholipid metabolism, which are not well understood in the conifer SEs. Here, we integrated lipidomics, transcriptomics and proteomics analyses to reveal the molecular basis of lipid remodeling under PDT in *Picea asperata* SEs. Among the 82 lipid molecular species determined by mass spectrometry, phosphatidic acid (PA) had a significant effect after PDT and was the most critical lipid in the response to PDT. The transcriptomics results showed that multiple transcripts in the glycerolipid and glycerophospholipid metabolism pathways were differentially expressed, and these included five *PLDα1* transcripts that catalyze the conversion of phosphatidylcholine (PC) to PA. Furthermore, the enzyme activity of this phospholipase D (PLD) was significantly enhanced in response to PDT, and PDT also significantly increased the protein level of *PLDα1* (MA_10436582g0020). In addition, PA is a key factor in gibberellin, abscisic acid and ethylene signal transduction. One *GDI1*, one *DELLA*, three *ABI1s*, two *SnRK2s*, one *CTR* and 12 *ERFs* showed significantly differential expression between SEs before and after PDT in this study. Our data suggest that the observed increases in the PA contents might result from the activation of PLDα by PDT. PA not only affects the physical and chemical properties of the cell membrane but also participates in plant hormone signal transduction. Our work provides novel insight into the molecular mechanism through which PDT promotes the germination of SEs of coniferous tree species and fills the gap in the understanding of the mechanism of somatic embryo lipid remodeling in response to PDT.

## 1. Introduction

Somatic embryogenesis, which can store good germplasm almost indefinitely in cryopreservation, is the most promising large-scale asexual propagation technique for some economically and ecologically important coniferous tree species [1]. However, the efficiency of somatic embryogenesis technology in industrial applications is seriously restricted by low germination/conversion [2]. A prerequisite for the successful germination of spruce somatic embryos (SEs) is mild or partial desiccation treatment (PDT) [3,4,5]. The positive effects of PDT on SE germination have been attributed to the decrease in endogenous abscisic acid (ABA) levels and sensitivity [3,6,7]. The carbohydrate and storage protein content in SEs have been showed to be altered during PDT [8,9]. In addition, PDT enhanced the activity of adenine and uridine salvage enzymes. These enzymes participate in nucleotide synthesis, which is required for the initiation of SE germination [10]. However, PDT results in a drastic water deficit. The water content of SEs was shown to decline 76.28% on the first day of PDT [11]. Lipids are sensitive to changes in water content [12]. On the one hand, structural lipids, such as glycerolipids and glycerophospholipids, are the major components in cell membranes. Cell membranes are the main parts of the response environment. On the other hand, signaling lipids mediate various physiological responses to stimuli under stressful conditions.

Phospholipids, also known as glycerophospholipids, are a class of lipids with a glycerol backbone. One of the basic classes of phospholipids is phosphatidic acid (PA), which has only a phosphate group [13]. The biosynthesis of PA occurs primarily in the endoplasmic reticulum, where a fatty acid chain is transferred from acyl-CoA to lysophosphatidic acid (LPA), resulting in the formation of PA [14]. By adding choline, serine, ethanolamine, inositol or glycerol molecules, the phosphate head group can be further modified to different classes of phospholipids, such as phosphatidylcholine (PC), phosphatidylserine (PS), phosphatidylethanolamine (PE), phosphatidylinositol (PI) and phosphatidylglycerol (PG) [15]. These amphiphilic molecules are key structural components in cell membrane bilayers and cell signaling messengers.

PC and PE are structural lipids with very high contents in the membrane [16,17]. The plant membrane is at the foremost position to protect the plant from abiotic stresses, such as freezing, mechanical wounding and drought. Maintaining the stability and integrity of the cell membrane is the basis of regular cell metabolism and physiological processes. The lipid composition and content of the cell membrane directly affect the cell membrane structure stability and fluidity [18]. The physiological significance of phospholipids has been investigated in herbaceous plants. Plants have evolved the ability to actively regulate lipid components to adapt to environmental stress. Gasulla et al. [19] found that in *Craterostigma plantagineum*, the total lipid content was not changed under dehydration. However, the lipid composition was changed significantly. In Arabidopsis, PC is greatly degraded, while PE, PG, PS and PI are less degraded in response to freezing [20]. In the leaves of winter wheat seedlings, PC, PE and PG are primary degradation targets in response to PEG-induced water stress. Thus, PC is degraded to the largest degree [21]. In addition, two classes of galactolipids, monogalactosyl-diacylglycerol (MGDG) and digalactosyl-diacylglycerol (DGDG), are the major components in plastidic membranes, and their biosynthesis may be important for plant water tolerance [22,23].

PA, a signaling lipid, unlike structural lipids, can be accumulated and degrade transiently [24]. Various phospholipases, lipid kinases and phosphatases are required for their fast turnover [25]. PA can be generated via two distinct phospholipase pathways: (I) hydrolyzing structural phospholipids (e.g., PC and PE) by phospholipase D (PLD) or (II) phosphorylating phospholipase C produced diacylglycerol by diacylglycerol kinase [26]. PA is a key mediator in the signal transduction pathways of hormones and other biologically active compounds that ensure plant growth, development and resistance to adverse environmental factors. Regulatory signals originating from hormones, such as auxin, brassinosteroids, salicylic acid, ABA, jasmonic acid, ethylene (ETH) and gibberellins (GA), have been found to implicate PA as a second messenger [27]. PA regulates the activity of binding proteins by recruiting target proteins, such as protein kinases, phosphatases and vesicular transport proteins, to the plasma membrane or by directly inducing conformational changes [28]. In Arabidopsis, PA mediates the tethering of ABI1 (a negative regulator of ABA) to the plasma membrane to prevent it from reaching its downstream targets in the nucleus [29]. PA could bind to GA receptor OsGID1 to promote its nuclear localization for gibberellin response in rice [30]. PA is also involved in the process of ETH signaling by binding to CTRI, the negative regulator in the ethylene signaling pathway, and inhibiting its activity. PA inhibits its interaction with the ethylene receptor ETR1 [31].

Spruce is an important industrial timber tree in the world. Its straight trunk, light and soft wood make it the main raw material for construction, instruments, furniture, paper and wood fiber industry [32,33,34]. In addition, its root, stem and leaves are potential sources of aromatic oils, turpentine and tannin extracts [35]. Therefore, spruce trees have important commercial value. *Picea asperata* Mast is a Chinese endemic species that is one of the most widely distributed *Picea* species in China [36]. We constructed a complete somatic embryogenesis system of *P. asperata* and a somatic embryo germination technique induced by PDT [11]. In this study, highly synchronized SEs from the embryogenic cell line 1931 were used for lipidomics, transcriptomics and proteomics analyses to explore (I) the changes in membrane lipids during PDT, (II) the key lipids that change in response to PDT and (III) the potential regulatory mechanism of the membrane lipid response to PDT. This work provides novel insight into the molecular mechanism by which PDT promotes SE germination from the perspective of membrane lipid remodeling.

## 2. Results

### 2.1. Lipid Profiles Significantly Changed in SEs of P. asperata after PDT

SEs of *P. asperata* were collected before and after 14 days of PDT (D0 and D14). The lipid profiles of 82 lipid molecular species were determined by mass spectrometry (Table S1). Specifically, the composition and content of nine phospholipid classes (PG, PC, PE, PI, PS, PA, LPC, LPE and LPG) and two galactolipid classes (MGDG and DGDG) were determined.

**Figure 1:**
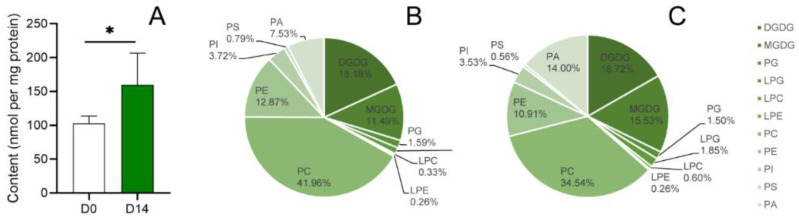
The total content and proportion of lipid components on *P. asperata* SEs. (**A**): The total lipid content of SEs before and after PDT. * Indicates a 5% significant difference. (**B**): The proportion of lipid components of SEs before PDT. (**C**): The proportion of lipid components of SEs after PDT for 14 days.

The total lipid content was higher in the D14 group than in the D0 group (*p* < 0.05) (Figure 1A). Proportion analysis of the lipid metabolites (Figure 1B,C) showed that the proportions of PA, PC and MGDG changed by more than 4% between D0 and D14. Specifically, the proportion of MGDG and PA increased by 4.04% and 6.47%, respectively, after PDT. The proportion of PC decreased significantly (from 41.96% to 34.54%). The proportions of PE and DGDG decreased from 12.87% and 18.18% to 10.91% and 16.72%, respectively, after PDT. The proportions of PS, PI, PG, LPC, LPE and LPG changed nonsignificantly after PDT (less than 1%).

**Figure 2:**
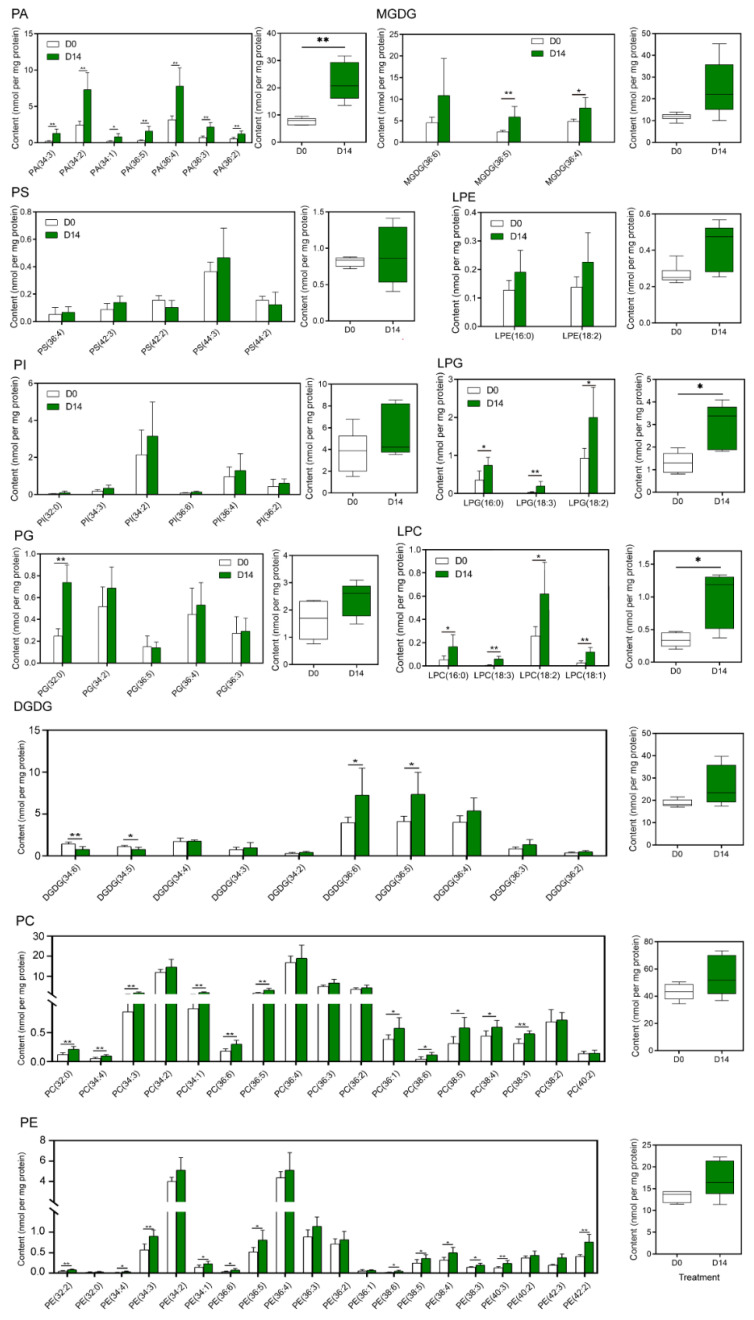
Changes in the levels of glycerolipid and glycerophospholipid molecular species in SEs subjected to PDT. * Indicates a 5% significant difference. ** Indicates a 1% significant difference. Values are mean ± SD (*n* = 6).

Phospholipids, crucial components of cell membranes, include PA, PC, PE, PG, PS and PI. Most phospholipid levels increased after PDT. A significant difference was detected in the total content of PA between D0 and D14. The total content of PAs increased by 187.87% after PDT. Furthermore, all seven molecular species of PAs increased significantly after PDT (Figure 2). In particular, the PA (34:2) and PA (36:4) levels in the D14 group were significantly increased by 196.30% and 145.59%, respectively, compared with those in the D0 group. On the other hand, the contents of PC (32:0), PC (34:4), PC (34:3), PC (34:1), PC (36:6), PC (36:5), PC (36:1), PC (38:6), PC (38:5), PC (38:4) and PC (38:3) in the D14 group were significantly increased by 59.94~84.31%. Twelve molecular species of PE were also significantly more abundant in the D14 group than in the D0 group. Lysophospholipids are the degradation products of phospholipids. The contents of four LPCs and three LPGs were significantly increased in SEs after PDT, and the increase in total LPC and LPG was also significant. MGDG and DGDG, belonging to galactolipids, are the major components of plastidic membranes. The contents of MGDG (36:5) and MGDG (36:4) at D14 were 2.43-times and 1.64-times higher than those at D0, respectively. Similarly, the contents of DGDG (36:6) and DGDG (36:5) were significantly higher on D14 than D0. Nevertheless, the contents of DGDG (34:6) and DGDG (34:5) were significantly decreased on D14 compared with those on D0.

### 2.2. Selection of Key Lipid Metabolites

**Figure 3:**
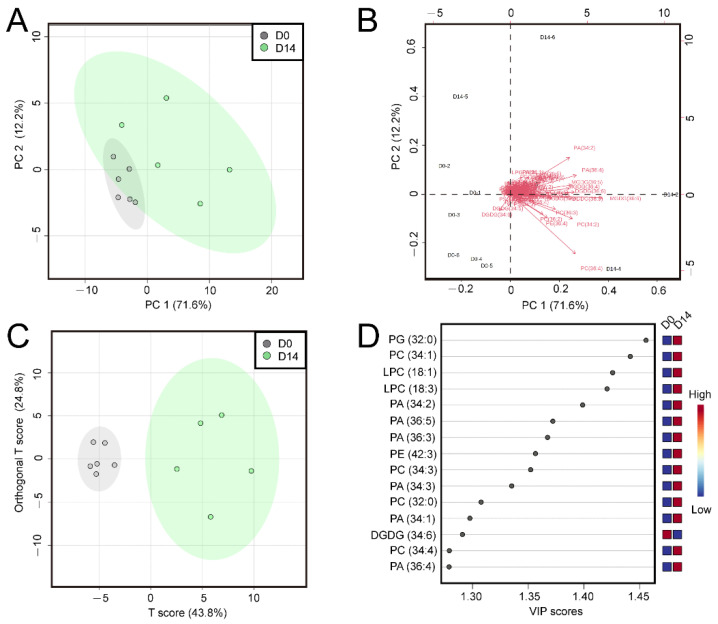
PCA and OPLS-DA of *P. asperata* SEs. (**A**): Score plots of the PCA. (**B**): Loading plots of the PCA. (**C**): Score plots of OPLS-DA. (**D**): VIP values of lipid molecular species.

Principal component analysis (PCA) was performed on the lipid profiles of D0 and D14 samples to explore the key lipids than changed in response to PDT (Figure 3A). The data showed that the six biological duplicates of D0 samples clustered together. This result indicated highly similar lipid profiles of the SEs before PDT. The six biological replicates of D14 samples were relatively dispersed. These results suggested dramatic changes in lipid composition or content in the SEs after PDT. In the biplot of the principal components (Figure 3B), the metabolites PC (36:4), PC (34:2), MGDG (36:6), PA (36:4) and PA (34:2) were relatively far from the origin of coordinates, which indicated a stronger power of identification between the compared groups. In addition, orthogonal partial least squares-discriminant analysis (OPLS-DA) was conducted to further analyze the key differential lipids between the D0 and D14 groups (Figure 3C). The variable importance in projection (VIP) value can reflect the importance of the independent variable X in interpreting the dependent variable Y in the model; usually, VIP > 1 is the threshold value: the greater the VIP value is, the greater the influence of this variable on the difference between groups. In this study, the VIP values of 15 lipid molecular species were greater than 1.25 (Table S2), including six PAs: PA (34:2), PA (36:5), PA (36:3), PA (34:3), PA (34:1), PA (36:4) and four PCs: PC (34:1), PC (34:3), PC (32:0), PC (34:4) (Figure 3D). Taken together, these data implied that PC and PA, especially PC (36:4), PC (34:2), PA (36:4) and PA (34:2), might be the main lipids involved in the physiological responses induced by PDT in *P. asperata* SEs.

### 2.3. Glycerolipid and Glycerophospholipid Metabolism Pathways Were Enriched in the Transcriptomes of SEs before and after PDT

To characterize the main regulators that may be involved in the metabolism of lipids during the PDT of the SEs of *P. asperata*, 20512 transcripts of SE cotyledons before PDT (CD0), 20802 transcripts of SE radicles before PDT (RD0), 21867 transcripts of SE cotyledons after 14 days of PDT (CD14) and 22032 transcripts of SE radicles after 14 days of PDT (RD14) were downloaded from PICEAdatabase (Lu et al., 2019). According to the transcript expression analysis (Figure S1), 4244 and 1756 transcripts were upregulated and downregulated, respectively, in CD0 vs. CD14. In total, 3413 transcripts were significantly upregulated and 1580 transcripts were significantly downregulated in RD0 vs. RD14. Differentially expressed genes (DEGs) were analyzed using the Kyoto Encyclopedia of Genes and Genomes (KEGG) database to identify those involved in lipid metabolic pathways.

**Figure 4:**
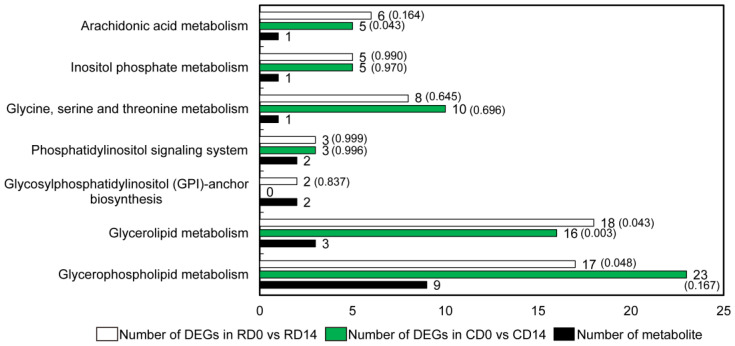
KEGG pathways involved in lipid metabolism enrichment of DEGs in CD0 vs. CD14 and RD0 vs. RD14. The values in parentheses are *p* values. The enrichment results of the former were significant at the 5% level.

According to KEGG pathway enrichment analysis, the 11 classes of lipids detected in this study involve a total of seven metabolic pathways, with the glycerolipid and glycerophospholipid metabolism pathways being enriched with three and nine detected lipids, respectively (Figure 4). DEGs were significantly enriched in both pathways. In CD0 vs. CD14 and RD0 vs. RD14, the glycerolipid metabolism pathway was significantly enriched in 18 and 16 DEGs, respectively. Meanwhile, in CD0 vs. CD14 and RD0 vs. RD14, 17 and 23 DEGs were significantly enriched in the glycerophospholipid metabolism pathway, respectively. These findings suggested that glycerolipid and glycerophospholipid metabolism played significant roles in the stimulus response induced by PDT in *P. asperata* SEs.

**Figure 5:**
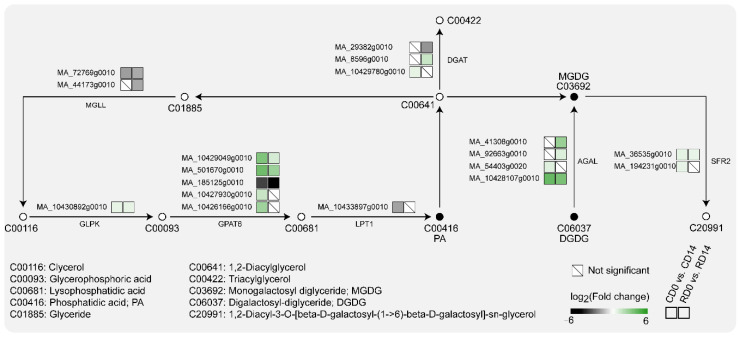
DEGs involved in glycerolipid metabolism pathways of desiccation-mediated SEs.

Glycerol-3-phosphate 2-o-acyltransferase (*GPAT6*) esterifies acyl groups from glycerophosphoric acid to lysophosphatidic acid in the glycerolipid metabolism pathway. Five and three transcripts encoding GPAT6 were found to be significantly altered in CD14 and RD14, respectively (Figure 5). Among these, MA_185125g0010 was significantly downregulated in both CD14 and RD14, with log_2_(FC) values of −3.04 and −4.34, respectively. The transcript levels of MA_10429049g0010 and MA_501670g0010 were increased by more than 10- and 3-fold in CD14 and RD14, respectively. Furthermore, the transcript levels of four *AGAL* genes, which catalyze the synthesis of MDDG from DGDG, were significantly upregulated in both CD14 and RD14. Among them, MA_10428107g0010 was upregulated 19.34-times and 14.34-times in CD14 and RD14, respectively.

**Figure 6:**
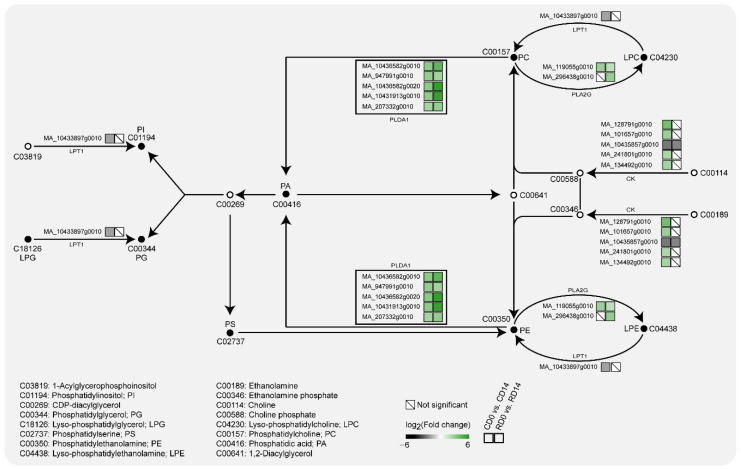
DEGs involved in glycerophospholipid metabolism pathways of desiccation-mediated SEs.

In the glycerophospholipid metabolism pathway (Figure 6), five *PLDα1* transcripts, which hydrolyze PC or PE at the terminal phosphodiesteric bond to generate PA, were significantly upregulated in CD14 and RD14. Their expression levels in CD0 vs. CD14 and RD0 vs. RD14 were both significantly upregulated by more than six-times. It is worth noting that MA_10431913g0010, MA_10436582g0010 and MA_10436582g0020 were significantly upregulated by 9.95, 10.15 and 10.69-times in CD0 vs. CD14, respectively, and these increases were more significant in radicles than in cotyledons, which were 46.22, 22.23 and 47.92-times higher in RD0 vs. RD14, respectively (Table S3). In addition, the transcription of *PLA2G*, which catalyzes the generation of LPC/LPE from PC/PE, was significantly upregulated in SEs after PDT. No transcriptional changes could be found in the phosphatidate phosphatase-encoding transcripts, which catalyze PA dephosphorylation to 1,2-diacylglycerol to produce PC and PE, in these transcriptomes. This result implied that the change in PA content mainly came from the hydrolysis of PC and PE.

**Figure 7:**
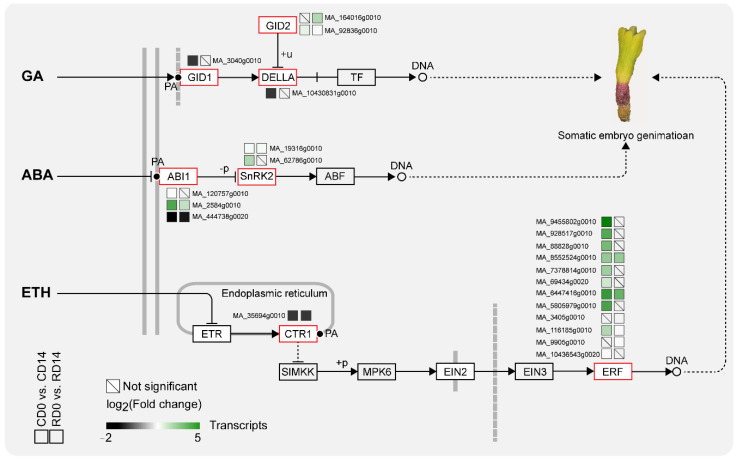
The DEGs involved in the plant hormone signal transduction pathway.

PA is a mediator in hormone signal transduction pathways. GA, ABA and ETH signals are involved in seed dormancy and germination. This study showed that the genes of the plant hormone signal transduction pathways changed significantly in SEs after PDT (Figure 7). Among them, the transcriptional levels of gibberellin receptors *GID1* (MA_3030g0010) and *DELLA* (MA_10430831g0010) were significantly downregulated in cotyledons after PDT. The three *ABI1*, a kind of protein phosphatase, transcripts were significantly differentially expressed in SEs after PDT, including MA_120757g0010 and MA_2584g0010, which were upregulated, and MA_444738g0020, which was downregulated. Notably, *SnRK2*, a downstream target gene of *ABI1*, was significantly upregulated. Similarly, it was found that the transcription of *CTR1* (MA_35694g0010) was downregulated in SE cotyledons and radicles after PDT. Twelve ERF transcripts, the downstream genes of the ethylene signal transduction pathway, were significantly upregulated in cotyledons or radicles after PDT; notably, MA_6447416g0010 was upregulated 10- and 7-fold, respectively, in the cotyledons and radicles. Studies have shown that PA can bind GID1, ABI1 and CTR proteins to participate in plant hormone signal transduction. We hypothesized that PDT promotes PA production and that PA further binds GID1, ABI1 and CTR to regulate downstream gene (*DELLA*, *SnRK2* and *ERF*) expression and promote somatic embryo germination.

### 2.4. The Enzyme Activity and Protein Level of PLD Were Significantly Increased after PDT

**Figure 8:**
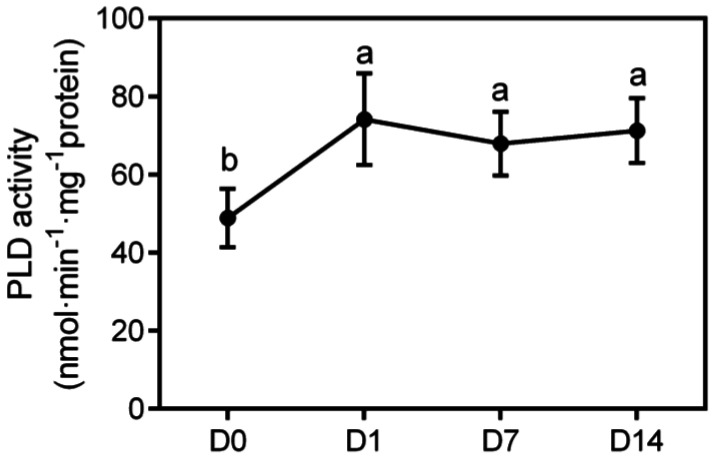
PLD activity of *P. asperata* SEs during desiccation. Different letters indicate 5% significant differences. Values are mean ± SD (*n* = 5).

Given that PLD-mediated hydrolysis makes the greatest contribution to PA formation during drought, we examined PLD activity to explore the reason why SEs maintain high levels of PA. Transcriptomic results showed that the expression of multiple *PLDα1* genes in SEs were significantly upregulated after PDT. To clarify the role of PLD in PDT, we measured the activity of the PLD enzyme before and after 1, 7 and 14 days of PDT (Figure 8). The activity of PLD in SEs increased significantly on the first day of PDT and then remained stable until the end of the experiment. The duration of desiccation was not the key factor for the change in PLD activity, but the presence of desiccation could significantly change PLD enzyme activity. This result suggested that PLD gene upregulation after PDT may increase the content of the PLD enzyme, and finally, increase enzyme activity.

To confirm our hypothesis, we analyzed the proteomic data of SEs after PDT, in which 150 and 275 proteins were upregulated and downregulated, respectively, in D0 vs. D14 [11]. The results showed that six differentially expressed proteins were involved in glycerolipid and glycerophospholipid metabolism and were significantly increased in SEs in the D14 group (Table 1). The protein levels of two PLDα enzymes (MA_10436582g0020 and MA_6712g0010) in the SEs of the D14 group were 5.31 and 1.39-times higher than the SEs of the D0 group, and the difference reached a statistically significant level. Interestingly, not only was the protein expression level of MA_10436582g0020 significantly increased, but its transcription level was also significantly increased in the SEs of D14. Taken together, we presume that MA_10436582g0020 is a key gene involved in the changes in phospholipid metabolism in response to desiccation for *P. asperata* SEs.

## 3. Discussion

PDT is an effective step to enhance the germination rate of SEs in conifer species. It has been reported that the SEs developed on the maturation medium are matured “morphologically” but not “physiologically” [37]. Desiccation, as a postmaturation treatment, is required for the “physiological” maturation of SEs [38]. Lipid metabolism, which participates in membrane reorganization and signal transduction, is one of the most important events during PDT. This metabolism occurs before other events and is a precondition for most cell processes.

The total lipid content of *P. asperata* SEs increased significantly after PDT (Figure 1). The lipid storage level of SEs is significantly lower than that of zygotic embryos in tamarillo (*Cyphomandra betacea*) [39]. Similar results were also found in a study of soybean SE germination [40]. These relatively low lipid levels are believed to be related to the high frequency of abnormal development of SEs when compared with zygotic embryos. Lipid storage can promote SE transformation to plants [41], and a higher lipid content could promote the dehydration tolerance of embryos and, consequently, improve vitality after germination [42].

The 11 classes of lipids detected in this study involve seven metabolic pathways, which mainly focus on the glycerolipid and glycerophospholipid metabolism pathways (Figure 4). Moreover, a transcriptome analysis revealed that these two pathways have multiple DEGs. The contents of LPG and LPC increased significantly after PDT, and these increases are due to *LPT1* transcripts, which catalyze the conversion of LPG and LPC into PG and PC, and exhibit significantly downregulated expression after PDT. The expression of *PLA2G* transcripts, which catalyze the conversion of PC into LPC, was significantly upregulated after PDT. In contrast, this study found that several phospholipids did not show an obvious trend and their contents did not change significantly after PDT. The proportions of PC and PE both decreased. The proportions of PS, PI and PG were less than 5%, and their changes were less than 1%. Under PEG stress, the composition of membrane lipids in the leaves of winter wheat seedlings showed a dynamic change. The levels of PC, PE and PG first slightly increased, then decreased rapidly to a low level 2 days after stress, and remained nearly stable for the following 2 days. However, the levels of PS and PI did not show a clear downward trend [21]. This finding suggests that the response of lipids to abiotic stress may be time dependent. Because this study did not determine the lipid content of SEs at different PDT stages, whether the levels of PC, PE, PS, PI and PG change throughout the PDT process remains to be studied.

PCA and OPLS-DA (Figure 3) showed that PC and PA compounds, especially PC (36:4), PC (34:2), PA (36:4) and PA (34:2), are the major phospholipids that respond to PDT in *P. asperata* SEs. PA showed the most obvious changes in our study. Not only did the content of seven molecular species of PA increase significantly (Figure 2) but the proportion of PA also increased by 6.47% (Figure 1). In contrast, the proportion of PC decreased from 41.96% to 34.54% after PDT (Figure 1). It is common that abiotic stress can induce changes in membrane lipids, especially an increase in PA content. PA, with its cone shape, is a special membrane structure component [28]. Its continuous and massive existence in membranes is affected by drought or desiccation [43]. The membrane transforms from the lamellar phase to nonlamellar phases (hexagonal I or II) under water deficit [23,44]. The cone-shaped PA predisposes the membrane to form the hexagonal II phase. In addition, the relatively high level of PA content in the SEs after PDT could provide the basis for the change in membrane phase and phospholipid composition. For instance, PA has been shown to activate the MGDG synthase MGD1 [45]. The high ratio of MGDG in the chloroplast membrane is important for the formation of thylakoids [46]. In this study, the proportion of MGDG increased by 4.04% after PDT. Previous studies have shown that the level of photosynthesis-related proteins increased in SEs after PDT [11]. The increase in MGDG could promote the formation of chloroplasts in the cotyledons of SEs. A study showed that successful germination requires the plastidic lipid MGDG and DGDG contents to increase to allow plastids to transform into shoots [23]. The increase in PA may be an intermediate of regulating lipid metabolism in response to PDT [47].

PA from diverse origins notably originates from the PLD-mediated hydrolysis of extraplastidial PC. Plant PLDs can be subdivided into six classes: α (α1, α2 and α3), β (β1 and β2), γ (γ1, γ2 and γ3), δ, ε and ζ (ζ1 and ζ2) [48]. PLD could be activated under various abiotic stresses. Among them, PLDα1 plays a role in transpirational water loss and, thus, affects drought resistance at the level of the whole plant [29], while PLDδ is also activated in response to rapid dehydration [49]. In this study, five *PLDα1* transcripts were significantly upregulated after PDT in the transcriptomes of CD and RD. In particular, MA_10436582g0020 increased by 10.69-times in cotyledons and 47.92-times in radicles. On the other hand, this study did not find significant changes in the gene expression of other enzymes that catalyze the production of PA. The enzyme activity of PLD in SEs subjected to PDT was significantly higher than that in SEs not subjected to PDT. The protein level in SEs after PDT was 5.31-times higher than that in SEs before PDT. Thus, we believe that PLDα1 is activated to produce PA in response to PDT.

PLD-mediated PA formation mediates various phytohormone signaling processes [50]. In this study, two *SnRK2* transcripts downstream of *ABI1* in ABA responses were found to be significantly upregulated in SEs after PDT. PLDα1-derived PA has been shown to bind to ABI1 [29]. The binding of PA to ABI1 decreases PP2C activity and tethers ABI1 to the plasma membrane. These changes inhibit ABI1 function by reducing the translocation of ABI1 to the nucleus. In addition, we found that 12 *ERF* transcripts downstream of *CTR* were significantly upregulated in SEs after PDT. PA can also bind CTR protein and inhibit its activity, which negatively regulates the ethylene signal transduction pathway [31]. This result implied that PA may participate in the ABA and ETH signal transduction pathways by binding with ABI1 and CTR proteins and, eventually, affecting SE germination.

## 4. Material and Methods

### 4.1. Plant Material

**Figure 9:**
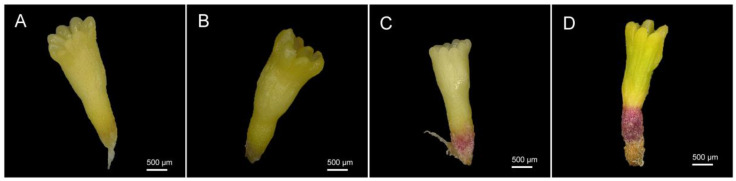
SEs of *P. asperata* after different PDT times. (**A**): without PDT, (**B**): desiccated for 1 day, (**C**): desiccated for 7 days, (**D**): desiccated for 14 days.

The plant materials used in this experiment were obtained by desiccating highly synchronized SEs from the embryogenic cell line 1931 [11]. The methods of cultivation and PDT are detailed below. Lipid content determination was performed on SEs desiccated for 0 and 14 days (D0 and D14). SEs desiccated for 0, 1, 7 and 14 days (D0, D1, D7 and D14) were collected for enzyme activity determination (Figure 9). All samples were collected in a cryopreservation tube, frozen in liquid nitrogen and stored at −80 °C for subsequent experiments.

### 4.2. Cultivation Conditions and PDT

After subculturing on semisolid medium for 14 days, filamentous adherent tissues were picked out at the edge of embryonic cell line 1931 with tweezers. Then, the cells were placed in an Erlenmeyer flask with 80 mL of liquid medium for suspension culture to quickly obtain many embryonic tissues [36]. Modified Litvay medium [51] with 10 μM 2,4-dichlorophenoxyacetic acid (Sigma, St. Louis, USA), 5 μM 6-benzylaminopurine (Sigma), 1% sucrose and 0.1% enzymatic hydrolyzed casein (Sigma) was used as the proliferation medium. Filter-sterilized glutamine (Sigma) was added to a final concentration of 0.05%. Then, the cells were cultured in the dark on a gyratory shaker at 110 rpm and 24 ± 1 °C. The vigorous embryonic tissue was transferred to fresh proliferation medium every 12 days. Mature SEs were obtained by transferring embryonic tissue with sterile filter paper to a differentiation medium. Using modified Litvay medium with 3% sucrose, 0.1% activated charcoal (Sigma), 61 μM filter-sterilized (±) cis, trans-ABA (Gibco-BRL, Gaithersburg, MD, USA), 5% polyethylene glycol 4000 (PEG4000, Merck, Darmstadt, Germany) and 0.4% gellan gum (Sigma) at pH 5.8. Filter-sterilized glutamine (Sigma) was added to a final concentration of 0.05%. The cultures were kept in the dark at 24 ± 1 °C for 7 weeks. Mature SEs with similar morphology were selected using the “filter paper” method to carry PDT [11,52]. The SEs were cultured under a 16 h photoperiod with a light intensity of ~15 μmol/m^2^/s (LED fluorescent tubes) for 2 weeks.

### 4.3. Transcriptomics, Proteomics and KEGG Pathway Enrichment of DEGs

Transcriptome and proteome data were obtained from the previous research basis of our group [53], and the detailed data were deposited into the PICEAdatabase (http://www.piceadb.com/) (accessed on 18 September 2020). In this study, the sequencing data of SE cotyledons and radicles with and without PDT for 14 days were used for subsequent analysis. We used the edge R package (http://www.rproject.org/) (accessed on 28 July 2021) to perform quasi-likelihood F tests. The DEGs had to meet the following conditions: fold change (FC) ≥ 2, *p* value < 0.05, and FPKM > 1.

KOBAS 2.0 (http://kobas.cbi.pku.edu.cn/kobas3/genelist/) (accessed on 27 August 2021) [54] was used to perform KEGG pathway enrichment analysis of DEGs from CD0 vs. CD14 and RD0 vs. RD14. Then, the *p* value was corrected by the Bonferroni method, and an adjusted *p* value (Q-value) ≤ 0.05 was taken as the threshold.

### 4.4. Lipidomics Analysis

SEs desiccated for 0 and 14 days were used for the determination of lipids. Each group of samples contained six biological replicates. The extraction method of total lipids referred to reference [20] and was modified as follows: The tissue samples were ground into powder in an ice bath and transferred to a centrifuge tube. A total of 500 μL of 2:1 chloroform/methanol was added, and the lipid extract was transferred to a glass tube with a screw cover. The above process was repeated 3 times. Then, 1 mL of 1 M KCl was added to the mixed extract and centrifuged for 20 min at 18,000× rpm. Finally, nitrogen was used to dry the organic phase, which was stored at −20 °C. A 4000 Q-Trap quadrupole mass spectrometer with an ion trap and electrospray ionization source (Applied Biosystems (China), China, Shanghai) was used to detect lipid compounds. The mass spectrometric results were processed according to reference [20].

PCA and OPLS-DA were performed using the online metabolism analysis database MetaboAnalyst 5.0 (https://www.metaboanalyst.ca/faces/home.xhtml) (accessed on 20 March 2022) [55]. Pareto scaling (mean-centered and divided by the square root of the standard deviation of each variable) was applied before data analysis.

### 4.5. Activities of PLD

The SEs after desiccation for 0, 1, 7 and 14 days were collected to measure enzyme activity. PLD activity was detected according to reference [56] as follows: (1) 1 mL of HEPES buffer (pH 7.0, 0.32 M sucrose, 1 mM dithiothreitol, 1 mM benzyl sulfonyl fluoride and 1 mM ethylene glycol tetraacetic acid) were added to approximately 0.1 g of SE sample. Then, the samples were centrifuged at 12,000× *g* for 45 min and the supernatant was taken and centrifuged at 15,000× *g* for 1 h. PLD crude extract was obtained by dissolving the precipitate in 1 mL of 100 mM pH 6.5 dimethylglutaric acid (DMG). The protein content was determined by the Coomassie bright blue method. (2) The 200 μL reaction system consisted of 0.1 mM DMG (pH 6.5), 10 mM MgCl_2_, 10 mM CaCl_2_, 5 mM linoleic acid and 50 μL PLD crude extract (control was replaced by DMG). Then, 12 mM phosphatidylcholine was added, sealed and heated at 30 °C for 30 min, followed by boiling in a water bath for 10 min to terminate the reaction. After cooling, 0.8 mL of solution (45 mM pH 8.0 Tris-HCl, 0.8 U choline oxidase, 2.4 U HRP, 0.24 mg 4-ATT and 0.16 mg phenol) was added and reacted at 30 °C for 90 min. Then, 1 mL of 45 mM Tris-HCl (contain 2 g/L Triton X-100, pH 8.0) was added after the color was stable. The protein was filtered with a 0.22 μm pore diameter filter and the absorbance was measured at 500 nm.

### 4.6. Statistical Analysis

SPSS 22.0 was used to measure significance with the independent-samples T test. GraphPad Prism 8 and Adobe Illustrator CS6 software were used to generate the figures.

## 5. Conclusions

PDT will increase the overall content of lipids and change the proportion of specific lipid components. The most significant increase was the increase in PA content. This phenomenon will affect the physicochemical properties of the cell membrane and lipid signal transduction. Combined with transcriptomic and proteomic data, it was found that PDT may activate *PLDα1* to promote the transformation of PC to PA. In addition, PA may further influence the process of somatic embryo germination by mediating the hormone signaling pathway. This study provides novel insight into the molecular mechanism by which PDT promotes the germination of SEs of coniferous tree species and fills the gap in the understanding of the mechanism of somatic embryo lipid remodeling in response to PDT.

## Figures and Tables

**Table 1:** Differentially expressed proteins involved in glycerolipid metabolism and glycerophospholipid metabolism in D0 vs. D14.

Protein ID	Corresponding Transcript ID	FC (D0 vs. D14)	KO ID	Uniprot Annotation
Pasi_116787465	MA_138834g0010	2.666 *	K12355	Aldehyde dehydrogenase 9 (Fragment)
Pasi_148910753	MA_10435526g0010	1.692 *	K14085	Aldehyde dehydrogenase family 7 member B4
Pasi_148906521	MA_9944g0020	1.315 *	K15918	D-glycerate 3-kinase, chloroplastic
Pasi_116786790	MA_97566g0010	1.635 *	K00128	Aldehyde dehydrogenase family 3 member H1
Pita_383143100	MA_10436582g0020	5.311 *	K01115	Phospholipase D alpha 2
Pasi_116787472	MA_6712g0010	1.393 *	K01115	Phospholipase D alpha 1

* Indicates a 5% significance level.

## Data Availability

Transcriptome and proteome data are available in PICEAdatabase (http://www.piceadb.com/).

## Supplementary Materials

The following supporting information can be downloaded at: https://www.mdpi.com/article/10.3390/ijms23126494/s1.

## Abbreviations

ABA	Abscisic acid
CD	Cotyledon by desiccation
DEGs	Differentially expressed genes
DGDG	Digalactosyl-diglyceride
ETH	Ethylene
FC	Fold change
GA	Gibberellin
KEGG	Kyoto Encyclopedia of Genes and Genomes
LPA	Lysophosphatidic acid
LPC	Lyso-phosphatidylcholine
LPE	Lyso-phosphatidylethanolamine
LPG	Lyso-phosphatidylglycerol
MGDG	Monogalactosyl-diglyceride
OPLS-DA	Orthogonal partial least squares-discriminant analysis
PA	Phosphatidic acid
PC	Phosphatidylcholine
PCA	Principal component analysis
PDT	Partial desiccation treatment
PDT	partial desiccation treatment
PE	Phosphatidylethanolamine
PG	Phosphatidylglycerol
PI	Phosphatidylinositol
PLD	Phospholipase D
PS	Phosphatidylserine
RD	Radicle by desiccation
SE	somatic embryo
SEs	Somatic embryos
VIP	Variable importance in projection
